# Targeting Transcription Factor YY1 for Cancer Treatment: Current Strategies and Future Directions

**DOI:** 10.3390/cancers15133506

**Published:** 2023-07-05

**Authors:** Rendy Hosea, Sharon Hillary, Shourong Wu, Vivi Kasim

**Affiliations:** 1 Key Laboratory of Biorheological Science and Technology, Ministry of Education, College of Bioengineering, Chongqing University, Chongqing 400044, China; 2 The 111 Project Laboratory of Biomechanics and Tissue Repair, College of Bioengineering, Chongqing University, Chongqing 400044, China; 3 Chongqing Key Laboratory of Translational Research for Cancer Metastasis and Individualized Treatment, Chongqing University Cancer Hospital, Chongqing University, Chongqing 400030, China

**Keywords:** yin yang 1 (YY1), YY1-targeted therapy, clinical implications, antitumor therapy, drug resistance

## Abstract

**Simple Summary:**

Cancer is a global health problem with severe consequences. Certain genes, known as transcription factors (TFs), are overactive in many tumors. Targeting these TFs could be an effective approach to combat cancer. One such TF is called yin yang 1 (YY1) and plays important roles in tumor development. In preclinical studies, inhibiting YY1 has shown promise in slowing tumor growth, promoting cell death, and increasing the effectiveness of chemotherapy. Recent research suggests that combining YY1 inhibition with immunotherapy may enhance the effectiveness of treatment. However, there are challenges in developing drugs that specifically target YY1 and delivering them into the tumor. This review explores YY1 biology, its role in cancer, and various strategies for targeting YY1, including small molecule inhibitors, RNA interference, and gene editing techniques. The findings highlight the clinical implications of YY1-targeted therapy and the potential for novel therapeutic approaches that can improve patient outcomes.

**Abstract:**

Cancer represents a significant and persistent global health burden, with its impact underscored by its prevalence and devastating consequences. Whereas numerous oncogenes could contribute to cancer development, a group of transcription factors (TFs) are overactive in the majority of tumors. Targeting these TFs may also combat the downstream oncogenes activated by the TFs, making them attractive potential targets for effective antitumor therapeutic strategy. One such TF is yin yang 1 (YY1), which plays crucial roles in the development and progression of various tumors. In preclinical studies, YY1 inhibition has shown efficacy in inhibiting tumor growth, promoting apoptosis, and sensitizing tumor cells to chemotherapy. Recent studies have also revealed the potential of combining YY1 inhibition with immunotherapy for enhanced antitumor effects. However, clinical translation of YY1-targeted therapy still faces challenges in drug specificity and delivery. This review provides an overview of YY1 biology, its role in tumor development and progression, as well as the strategies explored for YY1-targeted therapy, with a focus on their clinical implications, including those using small molecule inhibitors, RNA interference, and gene editing techniques. Finally, we discuss the challenges and current limitations of targeting YY1 and the need for further research in this area.

## 1. Introduction

Cancer is a pervasive and deadly disease, claiming nearly 10 million lives annually [1]. Despite significant progress in treatment options such as chemotherapy, radiation therapy, and targeted therapies, cancer remains a significant public health challenge with high morbidity and mortality rates [2,3]. Tumor development is driven by aberrant gene expression, leading to dysregulation of the signal transduction pathways that promote oncogenic growth. Although there have been improvements in the survival rates for certain types of cancer, others remain difficult to treat and have a poor prognosis. Consequently, there is an urgent need for novel therapeutic approaches that can improve patient outcomes [4]. Transcription factors (TFs) are key regulators of gene expression, and their dysregulation can affect multiple hallmarks of cancer. By modulating the activity of oncogenic signal transduction pathways and regulating tumor gene expression, TFs play a crucial role in tumor progression [5] as they are involved in many aspects of tumors, including oncogenic signal transduction, cell death resistance, and drug resistance. Targeting TFs, therefore, represents a promising antitumor therapeutic strategy that could modulate a broader range of tumor properties and thus achieve a more robust and sustained therapeutic response in contrast to the typically limited effects observed in various kinase inhibitors, which primarily only block specific signaling pathways in tumor cells such as epidermal growth factor receptor (EGFR) inhibitor, Bcr-Abl inhibitor, and receptor tyrosine-protein kinase erbB-2 (HER2) inhibitor. Notably, clinical agents that target nuclear hormone receptors, a class of transcription factors that are activated by binding to a specific hormone and are then translocated to the nucleus to perform their transcription factor activity, have shown promising results in tumor treatment [6,7,8].

YY1 is a multifunctional transcription factor that plays a critical role in regulating the expression of the genes involved in various physiological processes, including development, cell proliferation, differentiation, DNA repair, and apoptosis [9,10,11,12,13,14]. Formerly known as NF-E1, YY1 was named for its dual activity as both a transcriptional activator and repressor [15]. YY1 has attracted attention as a target for antitumor therapy due to its aberrant expression in various tumors and its wide range of target genes; this range is predicted to occupy approximately 7% of mammalian genes [16]. Furthermore, in addition to its role as a traditional DNA-binding transcription factor, YY1 interacts with chromatin modifications through 3D chromatin organization to regulate cellular mechanisms more broadly [17]. YY1 modulates a mounting list of genes in different signaling pathways that regulate tumor development and progression, such as c-myc, c-fos, HER2, E1A, and p53 [18,19], and is involved in regulating various hallmarks of cancer, including sustained proliferative signaling, evading of programmed cell death, and deregulated metabolism.

YY1 has emerged as a promising target for antitumor therapy in recent years due to its critical role in regulating various hallmarks of cancer, such as tumor cell proliferation, evading programmed cell death, deregulated metabolism, induction of angiogenesis, activation of invasion and metastasis, genome instability, and evading immune system [11,19,20,21], as well as in tumor cell drug resistance [22,23,24]. YY1 is upregulated in various human cancers, including breast, bladder, cervical, colon, esophageal, liver, brain, and gastric cancers, and there is increasing evidence suggesting that it has pro-tumor consequences (Table 1) [25,26,27,28,29,30,31,32]. Moreover, YY1 upregulation is associated with poor prognosis and aggressive tumor behavior, characterized by increased tumor growth, invasion, and metastasis [25,26]. Therefore, targeting YY1 has the potential to inhibit tumor progression and sensitize tumor cells to therapy.

As a target for antitumor therapy, several approaches have been explored to target YY1, including small molecule inhibitors, RNA interference, and gene editing techniques. Small molecule inhibitors that disrupt the interaction between YY1 and its DNA binding sites have shown promising results in preclinical studies by inhibiting tumor growth and metastasis in various types of cancer [33,75,76]. Knocking down *YY1* using RNA interference has also demonstrated efficacy in inhibiting tumor growth and sensitizing tumor cells to chemotherapy [31,33]. In preclinical studies, YY1 knockdown promotes apoptosis, inhibits cell proliferation, and enhances the effectiveness of chemotherapy in tumor cells. These findings suggest that targeting YY1 could be a promising strategy for tumor therapy. Additionally, gene editing techniques, such as clustered regularly interspaced short palindromic repeats (CRISPR)–CRISPR-associated (Cas) 9 (CRISPR-Cas9), have shown promise in preclinical studies as a means of targeting YY1 for antitumor therapy. Preclinical studies on gene editing techniques have demonstrated promising results in inhibiting tumor growth and metastasis in various types of cancer, including breast cancer, prostate cancer, and liver cancer [77,78,79]. However, clinical data on YY1-targeted therapies are currently lacking and further research, including clinical trials, is needed to fully demonstrate the safety and efficacy of YY1-targeted therapies in tumor patients. Nevertheless, the emerging evidence for the role of YY1 in cancer highlights its potential as a potential target for future research [33,56].

In this review, we will explore the potential of YY1-targeted therapy as a novel approach for tumor treatment and provide an overview of the different strategies that have been explored in preclinical and clinical studies.

## 2. The Roles of YY1 in Tumor Development and Progression

### 2.1. YY1 and Hallmarks of Cancer

Dysregulation of YY1 expression or function has been implicated in the pathogenesis of various types of cancer by influencing the hallmarks of cancer (Table 2) [19,20,80]. “The Hallmarks of Cancer” was originally proposed as a set of functional capabilities that human cells acquire during the transition from normal to neoplastic growth states [81,82,83]. These capabilities (e.g., sustaining proliferative signaling, insensitivity to growth-inhibitory signals, evasion of programmed cell death, limitless replicative potential, sustained angiogenesis, and tissue invasion and metastasis) are crucial for the formation of malignant tumors. Although this framework provides a solid foundation for understanding the biology of tumor cells, the original six hallmarks of cancer have since been updated to incorporate new developments that broaden the scope of cancer biology [81,82,83]. YY1 promotes tumor progression by regulating the expression of genes involved in cell proliferation and survival. YY1 activates the expression of oncogenes such as c-Myc, cyclin D1, and survivin and inhibits the expression of tumor suppressor genes such as p53 and p21 [61,84,85,86,87,88,89,90]. This leads to the dysregulation of the cell cycle and promotes the growth and survival of tumor cells [84,85,91,92,93].

YY1 can also promote angiogenesis, the formation of new blood vessels that supply nutrients and oxygen to tumors [84,103]. YY1 can stabilize hypoxia-inducible factor-1α (HIF-1α), thereby preventing its ubiquitination/proteasomal degradation and promoting transcription of the angiogenic factors vascular endothelial growth factor (VEGF) and transforming growth factor alpha (TGF-α) [84]. Furthermore, YY1 also promotes tumor angiogenesis by activating the transcription of VEGF while suppressing anti-angiogenic factors such as thrombospondin-1 (TSP-1), pigment epithelium-derived factor (PEDF), and tissue inhibitor of metalloproteinase 2 (TIMP-2) [103,133,134]. This leads to the formation of a network of blood vessels, thus supporting tumor growth and metastasis.

In addition, YY1 can promote tumor cell invasion and metastasis by regulating expression of the genes involved in cell adhesion and migration [135]. YY1 activates the expression of matrix metalloproteinases (MMPs) (enzymes that degrade the extracellular matrix), facilitating the remodeling of the surrounding tissue and creating the path for tumor cell migration. Simultaneously, YY1 inhibits the expression of E-cadherin, a protein crucial for cell–cell adhesion, further enabling tumor cell migration [43,110]. For instance, a study using gastric cancer cells showed that YY1 directly targets the *MMP-14* promoter and enhances its transcriptional activity. MMP-14 plays a crucial role in cell invasion; hence, when YY1-induced MMP-14 expression is suppressed by miR-584-3p through methylation of the YY1 binding site in the *MMP-14* promoter, the tumorigenesis and aggressiveness of gastric cancer cells were suppressed [43]. In addition, YY1 could promote epithelial–mesenchymal transition (EMT), the process at the initial stage of metastasis that is characterized by the loss of epithelial cell characteristics, including cellular polarity, cell-cell adhesion, and apical–basal polarity, as well as the acquisition of mesenchymal cell characteristics, such as increased migratory capacity, altered cytoskeletal organization, and enhanced extracellular matrix (ECM) remodeling [136,137]. EMT is characterized by the downregulation of genes that maintain the epithelial phenotype, such as E-cadherin, and the upregulation of genes that confer a mesenchymal phenotype, such as *Twist1*, *Snail*, and *Vimentin* [137]. YY1 activates the transcription of *Snail* and *Vimentin* by binding to their enhancer and promoter regions, respectively [55,138]. It also indirectly upregulates Twist1 by suppressing its inhibitor, heterogeneous nuclear ribonucleoprotein M (hnRNPM) [118]. Meanwhile, YY1 suppresses *E-cadherin* transcription by recruiting (protein arginine methyltransferase 7) PRMT7 to the proximal promoter of *E-cadherin*.

YY1 also suppresses the expression of genes involved in the immune response [139,140,141] and promotes immune evasion in tumor cells. Tumor cells may evade the host’s immune system through chronic inflammation, which inhibits and suppresses the function of effector immune cells, leading to tumor-promoting effects rather than tumor immunosurveillance. Additionally, tumor cells can create defects in antigen presentation mechanisms or upregulate ligands that neutralize cytotoxic T-cells, thereby eluding the adaptive immune system [98,142]. YY1 also contributes to immune evasion by activating the expression of immune checkpoint molecules such as programmed death-ligand 1 (PD-L1), which inhibits the activation of T cells and promotes immune tolerance in tumor cells [22].

### 2.2. YY1 and Drug Resistance

Drug resistance is a significant obstacle in the clinical therapy of cancer. Although chemotherapy drugs remain one of the most effective treatments for many types of cancer, the development of drug resistance can lead to the reduced effectiveness of these drugs and an increased risk of disease progression or recurrence [143]. Unfortunately, drug resistance is a common occurrence in many types of cancer and can contribute to treatment failure and poor patient outcomes [144].

**Figure 1:**
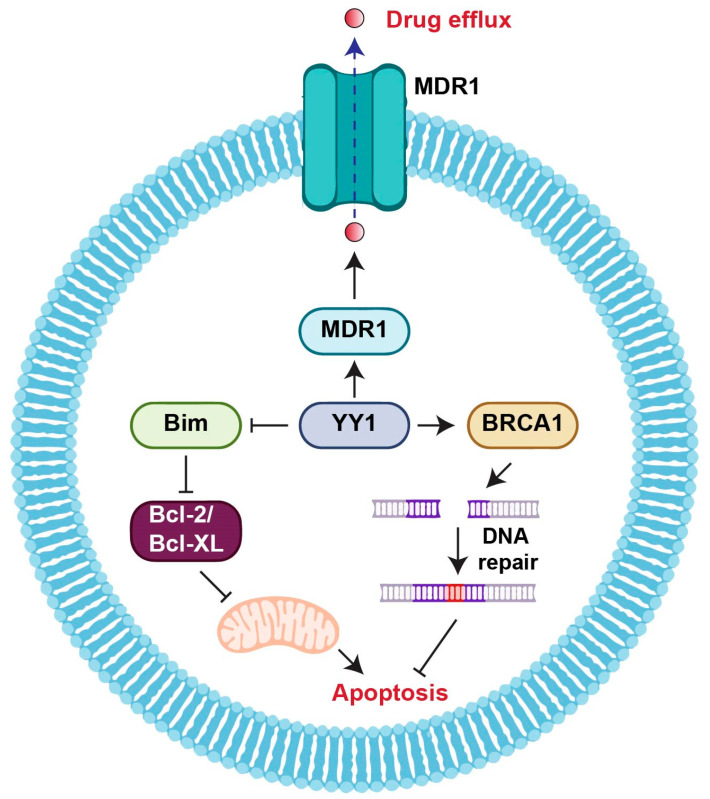
Schematic diagram illustrating the mechanism of YY1 regulation in tumor drug resistance. YY1 promotes drug resistance through regulation of the DNA repair response, anti-apoptotic proteins, and drug efflux transporters. Bcl-2: B-cell lymphoma-2; Bcl-XL: B-cell lymphoma-extra large; Bim: Bcl-2 interacting mediator of cell death; BRCA1: Breast cancer-associated gene 1; MDR1: multidrug resistance protein 1.

YY1 has been implicated in the development of drug resistance in tumor cells (Figure 1) [39,107,145]. It can activate the expression of multidrug resistance genes such as multidrug resistance protein 1 (MDR1) and multidrug-resistance-associated protein 1 (MRP1), which encode drug efflux pumps that remove chemotherapy drugs from tumor cells and reduce their effectiveness [51]. YY1 could also promote the survival of tumor cells in the presence of chemotherapy drugs by repressing anti-apoptotic genes such as Bcl2-interacting mediator of cell death (Bim) and increasing protein B-cell lymphoma-extra-large (BCL-xL) [57,96,104].

In addition, YY1 can regulate the expression of genes involved in DNA repair and cell survival in response to DNA damage [9,146]. YY1 activates the expression of breast cancer-associated gene 1 (BRCA1) and X-ray repair cross-complementing 1 (XRCC1), which repair DNA damage caused by chemotherapy drugs, thereby protecting tumor cells from apoptosis [90,147]. This leads to the development of drug resistance in tumor cells and reduces the effectiveness of chemotherapy drugs [90,147].

Emerging research has demonstrated that targeting YY1 may be a promising strategy to overcome drug resistance in antitumor therapy. Preclinical studies in prostate cancer cell lines have demonstrated that downregulating YY1 expression or inhibiting its activity can sensitize tumor cells to the chemotherapy drug cisplatin and enhance its effectiveness [148,149,150]. The combination treatment of cisplatin and DETA-NONOate reversed resistance and induced apoptosis in cisplatin-resistant prostate cancer cell lines. This chemosensitization occurred due to the inhibition of nuclear factor-kappa B (NF-κB), an upstream regulator of YY1, as well as the downregulation of downstream genes regulated by YY1, such as the anti-apoptotic genes Bcl-xL and XIAP [150]. In addition, some studies have suggested that YY1 inhibition may sensitize tumor cells to other therapies, such as radiation and immunotherapy [108,151,152]. Altogether, these findings highlight the importance of exploring novel approaches to overcome drug resistance in antitumor therapy.

### 2.3. YY1 and Cancer Stem Cells

Cancer stem cells (CSCs) are a subpopulation of tumor cells that have the ability to self-renew and differentiate into multiple cell types and are thought to be responsible for tumor initiation, progression, and recurrence [153,154]. Moreover, CSCs are closely linked to metastasis, drug resistance, recurrence, and poor prognosis, which contribute to the challenge of completely eliminating tumors [155]. YY1 has been implicated in the regulation of CSCs in several types of cancer [156]. High levels of YY1 expression in breast tumor samples have been reported to be associated with stem cell markers, such as Oct4, Sox2, and Nanog [156]. Additionally, overexpression of YY1 positively regulates the effect on the CSC phenotype by increasing various stemness traits, such as the expression of stem cell transcription factors, sphere-forming potential, the proportion of CD44^+^/CD24^−^ cells, and the ability to form tumors in vivo [157].

Notably, YY1 represses the expression of miR-879-5p by interacting with its promoter sequence, thereby activating the phosphatidylinositol 3-kinase/protein kinase B (PI3K/AKT) and extracellular signal-regulated kinase 1/2 (ERK1/2) pathways and, subsequently, maintaining the CSC phenotype [157]. In glioblastoma CSCs, YY1 mediates self-renewal through regulation of the SENP1/METTL3/MYC axis. YY1 transcriptionally upregulates sentrin/SUMO-specific protease 1 (SENP1) and enhances the methylase activity of methyltransferase-like 3 (METTL3), leading to increased N^6^-Methyladenosine (m6A)-modification levels in MYC mRNA, which promotes the self-renewal of glioblastoma stem cells (GSCs) [158]. These examples demonstrate that *YY1* overexpression leads to the maintenance of the CSC population, promotes tumor growth, and contributes to the resistance to therapy. Furthermore, YY1 can also regulate the interaction between CSCs and their microenvironment by activating the expression of cytokines and chemokines that recruit immune cells and promote the formation of a pro-inflammatory microenvironment that supports CSC survival and tumor growth [22,159].

Given the key role of CSCs in tumor initiation, progression, and recurrence, the targeting of CSCs has emerged as a promising therapeutic strategy for antitumor treatment. Many clinical trials targeting CSCs have been performed and show a promising future for antitumor therapy [155]. YY1 represents a potential therapeutic target in this context, given its key role in regulating CSCs in several types of cancer. For example, in brain CSCs, YY1 upregulates the expression of small nucleolar RNA host gene 17 (SNHG17), which extends the half-life of catenin beta 1 (CTNNB1) by sponging its direct negative regulator, miR-506-3p [160]. The upregulation of YY1 and SNHG17 promotes the activation of the Wnt pathway, which is associated with both tumorigenesis and CSC phenotype. Significantly, the downregulation of SNHG17 inhibits tumor growth in vitro and in vivo [160]. Additionally, YY1 inhibits miR-879-5p expression by interacting with its promoter sequence. This leads to the activation of the downstream PI3K/AKT and ERK1/2 pathways, which promote stemness in breast cancer cells. However, the suppressive effect of the YY1/miR-873-5p axis on the stemness of breast cancer cells can be reversed by inhibiting the PI3K/AKT and ERK1/2 pathways [157]. Therefore, understanding the mechanisms underlying YY1-mediated CSC regulation may provide novel therapeutic opportunities for antitumor treatment.

### 2.4. Current Development of YY1 Inhibitors

#### 2.4.1. Small Molecule Inhibitors of YY1

Small molecule drugs are chemical compounds that have the ability to interact with specific targets, such as proteins or DNA, and modify their function [161,162]. The advancement of modern molecular biology and the use of advanced technologies such as computer-aided drug design, structural biology, and combinatorial chemistry has facilitated the rapid development of small-molecule targeted drugs for antitumor therapy [161]. Currently, the FDA has granted approval to more than 89 small-molecule targeted drugs to treat different forms of cancer, and there are several thousand targeted agents undergoing clinical trials for antitumor therapy [8]. In comparison with macromolecule drugs such as monoclonal antibodies, polypeptides, antibody–drug conjugates, and nucleic acids, small-molecule targeted drugs offer several benefits in terms of their pharmacokinetic characteristics, affordability, patient adherence, and drug handling and transportation [161]. Small molecule drugs can target a wide range of proteins, including kinases, epigenetic regulatory proteins, DNA damage repair enzymes, and proteasomes [161]. Small molecule drugs have also attracted attention for antitumor therapeutic strategies targeting YY1. At present, there are several small molecule drugs that have been identified to influence YY1 protein activity either through direct or indirect inhibition [163].

#### 2.4.2. Diethylenetriamine NONOate (DETA-NONOate)

Nitric oxide donors can sensitize tumor cells to chemotherapy by releasing nitric oxide, which can alter the activity of various proteins containing sulfhydryl groups and modulate their activity through S-nitrosylation, which involves the transfer of a nitric oxide moiety to the sulfhydryl group of a protein [148,149]. One small molecule that has been investigated for its potential as a YY1 inhibitor is DETA-NONOate, which was reported in both in vitro and in vivo studies to inhibit the activities of YY1 and Bcl-xL, two proteins that can help tumor cells resist chemotherapy [150,164]. Treating tumor cells with DETA-NONOate resulted in the S-nitrosylation of YY1, leading to the inhibition of its DNA binding activity. Consequently, the negative regulation of Fas by YY1 was inhibited, resulting in upregulated Fas expression and tumor cell sensitization to Fas-induced apoptosis [150]. Moreover, animal studies have also shown that treatment with DETA-NONOate in combination with cisplatin led to a significant reduction in the expression levels of YY1 and Bcl-xL in tumor tissues [76,150,164]. Therefore, nitric oxide donors such as DETA-NONOate have great potential as antitumor therapy that targets YY1 and can enhance the cytotoxicity of antitumor therapies that depend on Fas-induced apoptotic tumor cell death, such as cell-mediated immunotherapy and immune checkpoint inhibition [165,166].

Although DETA-NONOate is still in the early stages of clinical development, nitric-oxide-mediated chemosensitization has shown potential as an antitumor therapeutic strategy in several clinical studies [148]. One example is nitroglycerin, which has shown promising results in lung cancer patients when used in combination with cisplatin, leading to improved response rates and longer time to progression [167]. Similarly, a clinical trial using slow-releasing nitroglycerine patches in men with high levels of prostate-specific antigen (PSA) after primary therapy resulted in a prolonged PSA doubling time, suggesting that nitric-oxide-induced inhibition of hypoxia-mediated progression may play a role in the observed therapeutic effects (NCT01704274) [168]. As a nitric oxide donor, nitroglycerin could inhibit NF-κB through S-nitrosylation, which could consequently interfere with NF-κB binding to DNA [169,170]. Another nitric oxide donor, RRx-001, is also capable of inducing NO production under hypoxic conditions and has demonstrated synergistic tumor cell cytotoxicity with radiation therapy by inhibiting the IκB kinase (IKK) complex, the master kinase for NF-κB activation [171,172,173]. NF-κB is an upstream regulator of YY1; hence, although the effect of nitroglycerin on YY1 has not been tested, these previous studies have suggested the possibility of using nitric oxide donors for an antitumor therapeutic strategy targeting YY1. However, NO donors may cause systemic toxicities, such as cytokine release syndrome. Therefore, there is a need for the development of new NO donors with localized effects to prevent systemic effects.

#### 2.4.3. Betulinic Acid

Betulinic acid, a triterpenoid derived naturally from tree bark extracts, possesses a wide range of pharmacologic properties, including antiviral, antibacterial, anti-inflammatory, antimalarial, and antitumor activities [174,175]. This compound causes significant growth inhibition of various tumors in animal models [175,176]. Its effectiveness as an antitumor drug can be attributed to its ability to induce mitochondrial toxicity and generate reactive oxygen species, leading to apoptosis in liver, bladder, and colon cancers. The efficacy of betulinic acid as an antitumor drug is attributed to its ability to induce mitochondrial toxicity and the production of reactive oxygen species, leading to apoptosis in liver, bladder, and colon cancers [177,178,179].

In one study, betulinic acid inhibited the growth of breast cancer cells through the downregulation of YY1 [75]. HER2 played a major role in the proliferation of breast cancer cells and was affected by betulinic acid treatment, resulting in decreased expression of HER2 and p-HER2, as well as downstream kinases such as mitogen-activated protein kinase (MAPK), p-MAPK, Akt, and p-Akt. It was demonstrated that the downregulation of HER2-regulated genes caused by betulinic acid is primarily attributed to the decreased expression of YY1, which acts as an upstream regulator of HER2 [180]. Furthermore, betulinic acid directly binds to cannabinoid receptors (CB) and disrupts the signaling pathway, leading to the disruption of the miR-27a repression of ZBTB10. This disruption, in turn, inhibits tumor growth in a xenograft model. Notably, ZBTB10 itself serves as a repressor of YY1. Thus, betulinic acid downregulates YY1 through a CB-dependent pathway and the miR-27a/ZBTB10 axis [75].

Furthermore, betulinic acid has also demonstrated the ability to inhibit tumor growth and inhibit lung metastases when used in combination with vincristine [181,182]. However, betulinic acid has more limited applications as an anti-cancer agent due to its poor solubility in aqueous media. To date, no clinical trials have been published evaluating the antitumor potential of betulin. Despite this limitation, betulin has a high potential for forming derivatives with better solubility and antitumor properties [183].

#### 2.4.4. ADP Ribosylation Factor like GTPase 6 Interacting Protein 5 (ARL6IP5) Gene Activating Compound (JAC1)

ARL6IP5, also known as JWA, is a gene that has been associated with the drug JWA activating compound 1 (JAC1). JAC1 is an antitumor drug that inhibits the proliferation of triple-negative breast cancer (TNBC) cells through the JWA/p38 MAPK and YY1/HSF1/p-Akt signaling pathways [184,185]. JAC1 specifically binds to YY1, thereby relieving YY1-mediated *ARL6IP5* transcriptional repression and increasing the expression of ARL6IP5 [184]. ARL6IP5 acts as a tumor suppressor gene in tumor cells and is associated with multiple functions, including angiogenesis, proliferation, apoptosis, metastasis, and resistance to chemotherapy [186,187,188]. Its downregulation in tumors is correlated with poor prognosis [184]. Treatment with JAC1 restores ARL6IP5 expression and induces G1 phase arrest and apoptosis in TNBC cells through the p38 MAPK signaling pathway. Furthermore, JAC1 not only promotes ubiquitination and degradation of YY1 but also disrupts the interaction between YY1 and heat shock factor 1 (HSF1), thus suppressing the oncogenic role of HSF1 in TNBC through the p-Akt signaling pathway [184]. Although further research and clinical trials are needed to fully understand the potential of JAC1 as an inhibitor of YY1, JAC1 could serve as a potential antitumor agent for *YY1*-overexpressed malignant tumors since YY1 is highly expressed in many cancers and regulates genes related to the cell cycle, cell death, and tumor metabolism.

#### 2.4.5. Peptide-Based Inhibition

In the past decade, advances in bioinformatics and genomics have uncovered a new class of small peptides called micropeptides, which are encoded by non-coding RNAs (ncRNAs). These micropeptides, typically composed of fewer than 100 amino acids, have been reported to play important roles in fundamental biological processes in a variety of organisms and may offer novel therapeutic opportunities that remain underexplored [189,190].

Micropeptides are distinct from other small bioactive peptides, such as neuropeptides and peptide hormones, which are typically derived from mRNA and are often cleaved from larger precursor proteins. Instead, micropeptides are encoded by small open reading frames (sORFs) within ncRNAs, such as long non-coding RNAs (lncRNAs) and circular RNAs (circRNAs) [191,192]. Recent genomic studies have helped to identify and characterize many novel micropeptides, which were often misannotated or overlooked. Some of these micropeptides have been found to contribute to diverse regulatory roles in embryogenesis, myogenesis, inflammation, diseases, and cancer [189,190,193].

Micropeptides have emerged as important regulators of cellular functions, including those involved in tumor cells. One lncRNA-encoded micropeptide that has garnered recent attention is the YY1-blocking micropeptide (YY1BM), which is encoded by *LINC00278* [194]. YY1BM specifically binds to the transcription factor YY1, blocking its interaction with the androgen receptor (AR) and leading to the downregulation of eukaryotic elongation factor 2 kinase (eEF2K) expression in tumor cells through the AR signaling pathway. This, in turn, induces apoptosis in esophageal squamous cell carcinoma (ESCC) cells, suggesting that YY1BM may have potential as an antitumor agent [194].

Recent studies have shown that low YY1BM expression is associated with reduced apoptosis in ESCC tumors and tissues and that YY1BM expression may be controlled by cigarette smoking in male ESCCs through the deletion of m6A modifications. These findings suggest that YY1BM may serve as a potential prognostic biomarker and therapeutic agent that specifically targets YY1 in tumors [194].

Besides micropeptides, synthetic peptides have been developed to disrupt YY1 oligomerization with oncoproteins such as enhancer of zeste homolog 2 (EZH2), mouse double minute 2 (MDM2), protein kinase B (AKT), and adenovirus early region 1A (E1A), an interaction critical for facilitating enhancer formation and subsequent gene expression. These peptides are designed based on the oncoprotein binding domain (OPB) sequence, a 26-amino-acid region between G201 and S226 of YY1 [195,196], and have shown promise in reducing the growth of xenograft tumors generated by TNBC cells by binding to either YY1 or EZH2 to disrupt the recruitment of EZH2 by YY1. This leads to reduced H3K27me3 at the phosphatase and tensin homolog pseudogene 1 (PTENP1) and its upregulation [197,198]. Micropeptides are still in early preclinical testing, and more research is needed to assess their clinical efficacy. Nevertheless, as shown by its specificity, targeting YY1 using micropeptides has great potential as an antitumor therapy, and these findings have attracted considerable attention in this field.

#### 2.4.6. Antibody-Based Inhibition

Several antibody-based inhibitors that affect YY1 have also been reported, including rituximab and galiximab. These drugs exert their antitumor effects by downregulating YY1 expression indirectly, which is achieved through the inhibition of NF-κB activity. NF-κB, which was previously known as the upstream regulator of YY1, is also a transcription factor that regulates immune responses and cell survival, and its dysregulation has been implicated in several human diseases, including cancer [199,200].

Rituximab, an FDA-approved monoclonal antibody targeting CD20, a protein expressed on B cells, has been used for treating B-cell non-Hodgkin’s lymphoma (B-NHL) and chronic lymphocytic leukemia (CLL) as it induces apoptosis [201,202]. Besides activating the caspase-dependent pathway, rituximab can downregulate the expression of YY1, which is upregulated in B-NHL and associated with poor prognosis, and inhibit NF-κB activity [62,203]. Rituximab decreased the phosphorylation of NF-κB-inducing kinase, IκB kinase (IKK), and IκB-alpha (IKK-α), as well as reducing the DNA binding activity of NF-κB, resulting in YY1 downregulation [203]. Rituximab also inhibits the chemoresistance mediated by NF-κB/YY1 axis regulation of Bcl-xL expression, thereby enhancing the efficacy of other antitumor drugs such as fludarabine, cyclophosphamide, and bendamustine. Furthermore, rituximab sensitizes B-NHL cells to immune-mediated killing by inhibiting NF-κB-mediated Fas resistance via YY1 downregulation [108,204].

Galiximab, a chimeric monoclonal antibody that targets CD80 and is currently in phase II clinical trials for the treatment of follicular lymphoma [205], could also downregulate YY1 expression by inhibiting NF-κB, which resulted in the induction of apoptosis in tumor cells [206]. In preclinical studies, galiximab has been shown to enhance the activity of cisplatin in terms of tumor cell killing [206,207], indicating that the combination of galiximab and cisplatin could be a promising strategy for the treatment of lymphoma.

#### 2.4.7. Nucleic-Acid-Based Inhibition

MicroRNAs (miRNAs) and lncRNAs are both non-coding RNA molecules that play a significant role in gene regulation by binding to the 3′ untranslated region (UTR) of messenger RNA (mRNA) and inhibiting its translation into a protein [208,209]. lncRNAs can regulate miRNA function by acting as competing endogenous RNA (ceRNA) to mediate miRNA sponging and alter expression levels and functions [209].

Recently, there has been increasing interest in using both miRNAs and lncRNAs as potential therapeutic agents for antitumor therapy [210]. One promising approach for miRNAs is to specifically target the transcription factor YY1. For example, miR-29a, which was previously reported to inhibit tumorigenicity in non-small cell lung cancer (NSCLC) by downregulating DNA methyltransferase (DNMT)3A and 3B, can suppress YY1 mRNA and protein expression levels in lung tumor cells, resulting in suppressed proliferation and migration [211]. Meanwhile, previous studies have revealed that miR-186 could downregulate YY1 expression in lung and prostate tumor cells through binding to complementary sequences at 3′ UTR region of YY1 mRNA, leading to decreased cell migration and invasion [212]. Several other miRNAs could also target YY1 in various cancer types, including miR-101, miR-181, miR-186, miR-193a-5p, miR-215, miR-218, miR-381, miR-544, miR-5590-3p, miR-635, miR-7, and miR-7-5p [40,74,95,211,212,213,214,215,216,217,218,219,220,221,222], demonstrating the potential of miRNAs in antitumor therapy strategies.

One example of the regulation mediated by lncRNA is nasopharyngeal carcinoma copy number amplified transcript-1 (NPCCAT1), which is overexpressed in nasopharyngeal cancer. NPCCAT1 interacts with the 5′-UTR of YY1 mRNA, increasing its translation and resulting in increased cell proliferation and migration [223]. Another example of the lncRNA regulation upon YY1 was reported in breast cancer. Expression of YY1 was indirectly increased by long intergenic non-protein coding RNA 958 (LINC00958), which was positively regulated by METTL3. LINC00958 functioned as a ceRNA to sponge miR-378-3p, a miRNA that targets YY1. Therefore, METTL3-mediated LINC00958 upregulation led to reduced miR-378-3p availability and increased YY1 expression that consequently enhanced tumorigenesis [224].

lncRNAs are druggable targets that can be modulated by antitumor agents. Furthermore, genetic tools such as small interfering RNA (siRNA) can be employed to regulate ncRNA expression in antitumor therapy [209]. The lncRNA–miRNA axis regulates cell death mechanisms such as apoptosis and autophagy in tumors [225,226]. Furthermore, the lncRNA–miRNA axis determines the sensitivity of tumor cells to various types of antitumor therapy including chemotherapy, radiotherapy, and immunotherapy [209].

Although promising results have been obtained from preclinical studies for both miRNA and lncRNA, there is currently no miRNA-based or lncRNA-based inhibitor targeting YY1 or YY1-regulating ncRNAs that has been developed for clinical trials. However, there are several nucleic-acid-based inhibitors that can selectively target miRNAs, such as antisense oligonucleotides (ASOs) and peptide nucleic acids (PNAs). ASOs can be designed to prevent the interaction of endogenous ncRNAs with their target genes by fully or partially complementing the non-coding RNA [227,228]. Similarly, PNAs could be used for recognizing RNA targets and inducing antitumor effects in vitro and in vivo. Nevertheless, various non-coding RNA (ncRNA)-based therapeutics for cancer are currently undergoing different phases of clinical trials [229]. For example, TargomiRs have undergone a phase 1 clinical trial (NCT02369198) to examine their tumor-suppressive effect as a second- and third-line treatment for patients with recurrent malignant pleural mesothelioma and non-small cell lung cancer [230]. Another ncRNA-based therapeutic, MRG106 (also known as cobomarsen), has been evaluated in a phase I clinical trial for patients with lymphoma and leukemia (NCT02580552) [231]. Furthermore, RNA therapeutics have received approval from the FDA and/or the European Medicines Agency for various diseases, including retinitis, hypercholesterolemia, muscular atrophy, amyloidosis, and hyperoxaluria [232].

There are several challenges to using ncRNAs to target YY1 in tumors. A major challenge is to selectively target YY1 while minimizing off-target effects. Another challenge is delivering ncRNAs to tumor cells. ncRNAs are rapidly degraded in the bloodstream and are not efficiently taken up by cells, so more efficient delivery systems are needed to specifically deliver ncRNAs to tumor cells [233,234].

One potential approach is to use such nanoparticle-based delivery systems that can protect ncRNAs from degradation and improve their uptake by cells [235]. However, these systems are still in development and their safety and efficacy need to be thoroughly evaluated. In addition, translation into clinical practice is a significant challenge as ncRNA-based therapies are still in the early stages of development and their safety and efficacy need to be examined further in clinical trials [236].

### 2.5. CRISPR/Cas9 Genome Editing of YY1

CRISPR/Cas9 genome editing is a revolutionary technology that allows precise and efficient manipulation of the genome. It involves using a guide RNA molecule to target a specific DNA sequence and the Cas9 protein to cleave the DNA at that site. This can lead to gene disruption, gene knockdown, or gene correction [77,78]. CRISPR/Cas9 offers several advantages over RNA-based drugs, such as higher precision, the ability to target multiple genes simultaneously, and the potential for long-lasting effects due to its ability to make permanent changes to the genome.

Recently a study by Xu et al. employed CRISPR/Cas9 to downregulate YY1 in prostate cancer [79]. YY1 directly binds to and activates phosphofructokinase (PFKP), a gene encoding a glycolytic rate-limiting enzyme that significantly promotes the Warburg effect [91,237,238]. The Warburg effect is essential for tumor cells to acquire the energy and metabolize the nutrients that enable synthesis of the macromolecular precursors necessary to support the malignant growth promoted by YY1 in advanced prostate cancer. Lowering YY1 expression reduces PFKP expression and tumor cell metabolism while inhibiting mitosis and promoting apoptosis of prostate cancer cells [79]. In vivo observations corroborate this finding, as xenograft tumors of *YY1* knockdown cells proliferate at a significantly slower rate than controls [79]. Although more preclinical studies need to be performed before CRISPR/Cas9 can be used in clinical settings, this result suggests that targeting YY1 expression using CRISPR/Cas9 offers promising potential for antitumor therapy.

### 2.6. YY1 and Immunotherapy

Combination therapy has emerged as a promising strategy for antitumor therapy, particularly in the context of immunotherapy. Although immunotherapy has shown remarkable success in some patients, many patients do not respond or develop resistance to therapy [239]. Therefore, combination therapy involving immunotherapy and other targeted agents, such as YY1-targeted therapy, has the potential to improve response rates and overcome resistance.

YY1 plays a crucial role in regulating the immune response in tumors by controlling the expression of various genes involved in immune cell activation, differentiation, and function in a range of immune cells, such as T cells, B cells, natural killer cells, and dendritic cells [101,121,139,140,141,240]. In immunotherapy, T cell exhaustion is a phenomenon that affects CD8^+^ T cells, where prolonged antigen exposure renders the cells hyporesponsive and incapable of eliminating tumor cells. This phenomenon is commonly linked to poor clinical outcomes in patients with solid malignancies [241]. Persistent antigenic stimulation causes the T cells to become exhausted, and it was reported that persistent T cell activation upregulates YY1 and EZH2 to epigenetically silence interleukin 2 (IL-2), a cytokine that plays a critical role in the activation and proliferation of T cells [241]. Therefore, the inhibition of YY1 has the potential to prevent T cell exhaustion and enhance the efficacy of immunotherapy by promoting IL-2 production and T-cell activation.

YY1-targeted therapy has good potential to be combined with immunotherapy drugs, such as checkpoint inhibitors and CAR-T cells. Checkpoint inhibitors are drugs that block inhibitory receptors on T cells, allowing them to better recognize and attack tumor cells [242]. However, many patients do not respond or develop resistance to checkpoint inhibitors [243]. In a previous study, the dual inhibition of cyclooxygenase-2 (COX-2) and EGFR by melafolone led to the downregulation of PD-L1, transforming growth factor beta (TGF-β), VEGF, and the PI3K/AKT pathway, which decreased tumor cell proliferation and enhanced the proliferation of CD8^+^ T cells [244]. This effect was likely due to the inhibition of PD-L1 expression, and it improved the efficacy of checkpoint blockade therapy [244]. Since YY1 is a positive regulator of both COX-2 and EGFR [101,111], inhibiting YY1 may be a promising strategy for increasing the effectiveness of checkpoint inhibitors. Therefore, combining YY1-targeted therapy with checkpoint inhibitors may have a synergistic effect on immune activation and warrants further investigation.

CAR-T cell therapy is a type of immunotherapy that involves engineering T cells to express chimeric antigen receptors that can recognize specific antigens on tumor cells. Whereas this approach has been successful in some patients, others do not respond or develop resistance to treatment [245]. Combining YY1-targeted therapy with CAR-T cells may help overcome resistance and improve response rates. One factor that can contribute to resistance is the expression of PD-L1 on tumor cells or the tumor microenvironment, which can inhibit CAR-T cell activity as these cells express programmed cell death protein 1 (PD-1) [245]. To address this, blocking immune checkpoints such as PD-1 may enhance the efficacy of CAR-T cell therapy. Previous studies combining anti-PD1 antibodies with CAR-T cells have shown promising results in a subset of patients [246,247,248,249]. Inhibition of YY1, which is a regulator of PD-L1 expression, maybe a promising strategy to overcome resistance in CAR-T cell therapy and improve patient outcomes.

**Figure 2:**
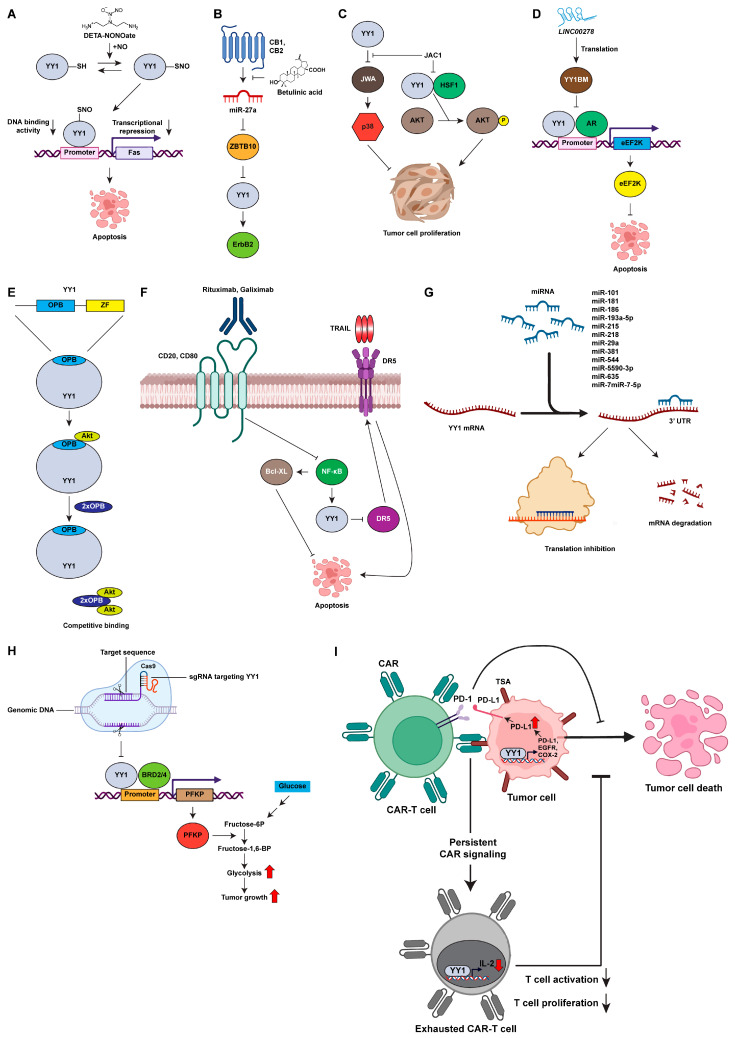
Targeting transcription factor YY1 for antitumor therapy. (**A**) DETA-NONOate inhibits YY1 binding to the promoter. (**B**) Betulinic acid inhibits YY1 through cannabinoid-receptor-dependent disruption of microRNA-27a:ZBTB10. (**C**) JAC1 targets YY1-mediated JWA/p38 signaling to inhibit tumor proliferation. (**D**) YY1BM inhibits the interaction between YY1 and the androgen receptor, which in turn decreases expression of *eEF2K* through the AR signaling pathway. (**E**) Synthetic peptides with the OPB domain disrupt the regulation of YY1 by competitive binding. (**F**) Antibody-based inhibition of YY1 through inhibition of the NF-κB signaling pathway. (**G**) Nucleic-acid-based inhibition of YY1. (**H**) CRISPR/Cas9 genome editing of YY1. (**I**) Role of YY1 in immunotherapy based on CAR-T cells. AKT: protein kinase B; AR: androgen receptor; BRD2/4: bromodomain-containing protein 2/4; CAR: chimeric antigen receptor; CAR-T cell: chimeric antigen receptor T cell; CB1: cannabinoid receptor 1; CB2: cannabinoid receptor 2; Cas9: CRISPR-associated protein 9; COX-2: cyclooxygenase 2; DETA-NONOate: diethylenetriamine NONOate; EGFR: epidermal growth factor receptor; eEF2K: eukaryotic elongation factor 2 kinase; ErbB2: erb-b2 receptor tyrosine kinase 2; Fas: Fas death receptor; HSF1: heat shock factor 1; IL-2: Interleukin 2; JWA: ADP ribosylation factor like GTPase 6 interacting protein 5 (ARL6IP5); LINC00278: Y-linked long noncoding RNA 278; NO: nitric oxide; OPB: oncoprotein binding domain; PD-1: programmed death 1; PD-L1: programmed death ligand 1; PFKP: phosphofructokinase, platelet; p38: p38 mitogen-activated protein kinase; sgRNA: single guide RNA; TSA: tumor-specific antigen; TRAIL: tumor necrosis factor related apoptosis-inducing ligand; YY1BM: YY1-blocking micropeptide; ZBTB10: zinc-finger and BTB domain containing 10.

Overall, these studies suggest that combination therapies involving YY1-targeted therapy and immunotherapy drugs have the potential to improve response rates and overcome resistance in antitumor therapy. Although clinical trials on drugs that directly target YY1 are still limited, clinical trials of certain drugs that may be correlated with the modulation of YY1 expression in cancer provide a strong foundation for the further development of a direct YY1 inhibitor. The strategies for YY1 inhibition discussed in this review are summarized in Figure 2 and Table 3.

### 2.7. Other Regulatory Functions of YY1

Besides its well-known function as a transcription factor, YY1 has also been reported to have functions beyond transcription factor roles. One such role is the post-translational modification of p53 by facilitating the interaction between MDM2 and p53 [88]. *YY1* overexpression stimulates p53 ubiquitination and degradation. Conversely, *YY1* silencing results in p53 accumulation due to a reduction in p53 ubiquitination and leads to increased tumor cell apoptosis. Furthermore, YY1 plays a role in stabilizing HIF-1α in response to hypoxic stress. Knockdown of *YY1* can reduce the accumulation of HIF-1α and its activity under hypoxic conditions, consequently downregulating the expression of HIF-1α target genes in p53-independent manner. This resulted in suppression of tumor cell proliferation and angiogenesis potential [84]. Additionally, epigenetic regulation is a hallmark of cancer and YY1 has been implicated in the epigenetic regulation of various genes involved in tumorigenesis [250]. Epigenetic modifications play a critical role in cancer development and progression by regulating gene expression without altering the underlying DNA sequence [250]. These modifications involve various mechanisms such as DNA methylation, histone modifications, and chromatin remodeling [250]. YY1 interacts with multiple chromatin modifiers, including the Polycomb complex [251,252], histone deacetylases (HDACs) [253], histone acetyltransferases (HATs) [254], and protein arginine N-methyltransferase 1 (PRMT1) [255]. These interactions allow YY1 to regulate gene expression through histone modifications. YY1 also interacts with chromatin remodeling complexes such as the INO80 complex and the BAF complex [9,256,257]. These interactions facilitate access of YY1 to target genes and enhance its binding to and regulation of those genes. In addition to its role in chromatin modification and remodeling, YY1 also plays a role in three-dimensional (3D) chromatin organization [258]. YY1 interacts with the proteins involved in chromatin organization such as CCCTC-Binding Factor (CTCF) and cohesion [259,260]. These interactions allow YY1 to regulate DNA loop formation within CTCF–CTCF domains. Together, these findings highlight the diverse roles that YY1 plays in cancer biology, from post-translational modification to epigenetic regulation. Therefore, YY1’s role in cancer has attracted significant interest and continues to be an active area of investigation.

**Table 3:** Summary of YY1 inhibitors and the results.

	Result	
YY1 Inhibitors	In Vitro	In Vivo
DETA-NONOate	Increased Fas-induced apoptosis [150]	Downregulated Bcl-xL expression in mice bearing PC-3 tumor xenograft [76]
	Sensitized cells to TRAIL-induced apoptosis in prostate cancer cell line (DU145, PC-3, CL-1, and LNCaP) [150]	Inhibited tumor growth [76]
RRx-001	Enhanced sensitivity to radiotherapy in HT29 and SCCVII cell lines [171]	Enhanced sensitivity to radiotherapy in mouse model [171]
		RRx-001 already passed phase I clinical trial. RRx-001 was well tolerated, with no notable toxicities nor adverse effects (NCT01359982) RRx-001 is currently in a phase 2 clinical trial [171,172,173]
	Inhibited Ikβ kinase complex [172,173]	Inhibited Iκβ kinase complex [172,173]
Betulinic acid	Downregulated YY1 in MDA-MB-453 cell line [75]	Downregulated YY1 in BT474 xenografted nude mice [75]
	Downregulated YY1-dependent HER2 expression in the MDA-MB-453 cell line [75]	Decreased tumor growth [75]
	Induced cell cycle arrest in G_2_/M phase [181]	Decreased β2-microglobulin mRNA [181]
	Decreased cell proliferation [180,181]	Inhibited tumor growth and metastases [181,182]
JAC1	Upregulated expression of ARL6IP5 [186,188]	Inhibited formation of neo-vessels in gastric-cancer-bearing nude mice [188]
	Downregulated HER2 expression [186]	Inhibited angiogenesis of melanoma [188]
	Reduced cell migration [186]	
YY1BM (LINC00278)	Downregulated eEF2K; induced apoptosis of ESCC cells [194]	Increased apoptosis [194]
Synthetic peptides (YPB and OPB)	Disrupted YY1-EZH2 [197]	Inhibited tumor growth in xenograft of MDA-MB-231 cells [197]
	Reduced H3K27me3 [197]	
	Upregulated PTENP1 and PTEN expression [197]	
	Inhibited cell proliferation of TNBC cell lines (MDA-MB-231 and MDA-MB-453) [197]	
	Reduced viability, reduced cell migration, in MDA-MB-231 [197]	
Rituximab	Inhibited NF-κB and Bcl-xL activity [108,203]	Increased tumor regression [108]
	Increased chemotherapy drug sensitivity [108,203]	
	Sensitized cells to immune-mediated killing [108,204]	
Galiximab	Inhibited NF-κB activity [207]	Chemosensitized malignant B cells [207]
	Reduced proliferation of B-NHL cell lines [207]	Galiximab already passed phase I/II clinical trial, result indicates that galiximab can be safely used. Galiximab is currently in phase III clinical trials [207]
	Sensitized resistant B cells to chemotherapy and immunotherapy [207]	
	Induced malignant B cell apoptosis [207]	
miR-29a	Downregulated DNMT 3A and 3B in A549 cells [211]	
	Suppressed cell proliferation and migration in A549 cells [211]	
	Inhibited IL-13-induced YY1 in A549 cells [211]	
	Inhibited tumorigenicity in A549 cells [211]	
	Decreased cell migration and invasion of A549 cells [211]	
miR-186	Inhibited proliferation, invasion, and migration of A549 and HCC827 cells [212]	
	Induced apoptosis of A549 and HCC827 cells [212]	
miR-181	Reduced cell proliferation of HeLa, HeLa-229, SiHa, and C33 cells [213]	Suppressed tumor growth in nude mice with HeLa cells [213]
	Increased cell apoptosis of HeLa, HeLa-229, SiHa, and C33 cells [213]	
miR-193a-5p	Decreased cell proliferation and migration of HEC-1-A, HEC-1-B, AN3CA, RL95-2, and KLE [95]	Inhibited development and progression of primary endometrioid endometrial adenocarcinoma [95]
miR-215	Suppressed cell proliferation, cell migration, and invasion in LS174T, LoVo, HT29, HCT116, SW480, and SW620 cells [214]	
miR-218	Inhibited cell proliferation of U251MG and 293T cells [220]	
miR-381	Inhibited cell proliferation, cell migration, and invasion of OVCAR3, Caov-3, OVCA429, SKOV3, A2780, and COV644 cells [217]	
miR-544	Decreased cell viability, proliferation, and migration of SW173 and 8350C [74]	Suppressed tumorigenicity of ATC cells [74]
miR-5590-3p	Inhibited cell proliferation and migration of MDA-MB-436, MDA-MB-468, BT549, and MDA-MB-231 [219]	Suppressed tumor growth xenograft mice model with BT549 cell [219]
miR-635	Inhibited invasion of H522 and H1299 cells [218]	Inhibited tumor growth in null mice with H522 cells [218]
miR-7	Suppressed cell proliferation of HCT116, LoVo, and DLD-1 cells [40]	Suppressed tumor growth in xenograft mice model [40]
	Induced apoptosis of HCT116, LoVo, and DLD-1 cells [40]	
miR-7-5p	Sensitized LN229 cells to temozolomide [222]	Sensitized LN229 cells to chemotherapy drug temozolomide in nude mice [222]
	Suppressed cell stemness of LN229 [222]	
TargomiRs		Already passed phase I Clinical trial against malignant pleural mesothelioma and NSCLC (NCT02369198) [230]; the result indicated that TargomiRs were well tolerated in the first 5 patients and associated with transient cytokine-mediated reactions.
MRG106 (Cobomarsen)		Cobomarsen already passed phase I clinical trial against lymphoma and leukemia (NCT02580552) [226]; the result indicated that cobomarsen was well tolerated, has potential clinical activity, and has the potential to improve the life quality of myelofibrosis patients.
CRISPR/Cas9	Reduced glycolysis of HEK293 and HEK293T cells [79]	Reduced cell proliferation in tumor xenograft of NOD/SCID/gamma null mice with 22Rv1 cells [79]
	Increased apoptosis of HEK293 and HEK293T cells [79]	

## 3. Conclusions

YY1-targeted therapies show promise as a novel approach to antitumor treatment; however, several challenges must be addressed before they can be translated into clinical settings. One of the challenges is the specificity of YY1-targeted therapies. Although small molecule drugs can decrease the expression of YY1, they may also affect other cellular processes, such as DNA replication and cell division, causing unintended consequences [161]. For example, studies investigating the effects of YY1 inhibition in tumor cells using a nitric oxide donor found that the drug not only inhibited YY1 expression but also caused cytokine release syndrome [148,261]. YY1 is a transcription factor that regulates the expression of many genes; targeting it may have unintended effects on normal cells and tissues. Therefore, it is essential to develop specific methods that can selectively target tumor cells while sparing normal cells. One potential approach is the use of antibody–drug conjugates (ADCs) for the selective delivery of YY1 inhibitors into tumor cells, thus minimizing the off-target effects on normal cells [262].

Moreover, another critical and fundamental problem is the high homology between YY1 and YY2, another member of the YY family of transcription factors [263]. YY1 and YY2 are highly homologous, with 56.2% similarity in their overall protein sequences and 86.4% similarity in the protein sequences within their zinc finger domains [264]. However, unlike YY1, which is upregulated in tumor tissues and is oncogenic, YY2 is downregulated in tumor tissues and is a tumor suppressor protein [93,263,265]. YY2 can trigger the ultraviolet damage response, p53-mediated cell cycle arrest, and tumor cell ferroptosis, thereby suppressing tumor growth [93,265,266]. Due to the high similarity in their nucleic acid and in amino acid sequences, designing drugs specifically targeting YY1 is challenging [264,267]. Furthermore, previous studies have reported the cross-reactivity of several antibodies targeting YY1 and YY2 [268]. These factors highlight the need for specific YY1 inhibitors that can selectively target YY1 without affecting YY2 to overcome the challenges posed by the homologous YY2 protein.

Furthermore, a cautionary note must be taken into account when developing and administering drugs targeting YY1. Although YY1 has oncogenic role in cancer, emerging reports suggest that it may also function as a tumor suppressor in certain cancer types [80]. The mechanism governing these opposing roles for YY1 is not yet fully understood. Therefore, it is crucial to consider the specific context of YY1 expression and activity to avoid unintentional adverse effects, such as the risk for secondary cancer. Careful assessment of the context-specific effects of YY1 modulation is necessary to ensure the safe and effective use of YY1-targeted therapies.

Another challenge is the efficacy of YY1-targeted therapies. Although they have shown promising results in preclinical studies, their efficacy in clinical settings may be limited by various factors, including drug resistance, the heterogeneity of tumor cells, and the tumor microenvironment [144]. To enhance their efficacies, developing combination therapies that target YY1 along with other pathways or drugs may be beneficial. Combination therapies can potentially overcome drug resistance, a major challenge in antitumor therapy, by targeting multiple pathways involved in tumor growth and progression [269]. For example, combining a YY1 inhibitor with an immunotherapy agent that targets the immune system’s response to tumors could enhance the immune system’s ability to recognize and destroy tumor cells, potentially improving patient survival [24,25].

Drug delivery to the tumor site is another challenge. The tumor microenvironment plays a crucial role in tumor progression and the treatment response and could impact the efficacy of YY1-targeted therapies in multiple ways [270,271]. For example, the presence of immune cells, such as T cells and myeloid-derived suppressor cells, can contribute to drug resistance by creating an immunosuppressive environment that shields tumor cells from the effects of treatment [270,271]. Moreover, the presence of stromal cells, such as cancer-associated fibroblasts, and extracellular matrix can hinder drug delivery to the tumor site by creating a physical barrier that prevents drugs from reaching the tumor cells [272]. Therefore, developing strategies to overcome these barriers and enhance drug delivery to the tumor site is essential to improve the efficacy of YY1-targeted therapies. Alternative drug delivery methods, such as nanoparticles or other drug delivery systems, have been explored to address this challenge [233,234,235]. These methods can improve drug delivery to the tumor site and potentially enhance therapeutic efficacy. For example, in a preclinical study, exosome-based nanoparticles could enhance the efficacy of delivering YY1 inhibitors into glioblastoma cells in both in vitro and in vivo models of the blood–brain barrier (BBB) [273]. Further research into alternative delivery methods for YY1-targeted therapies could offer new possibilities for antitumor therapy.

Finally, identifying biomarkers that can predict the response to treatment is a promising avenue for future research. Biomarkers, such as genetic mutations, epigenetic modifications, and protein expression patterns, have been reported to be associated with sensitivity or resistance to YY1-targeted therapies in preclinical studies [25]. By identifying patients who are most likely to respond to YY1-targeted therapies, personalized treatment plans can be developed to maximize therapeutic efficacy and minimize potential side effects [274]. Therefore, further research is needed to validate these biomarkers in clinical settings and to develop standardized tests for their detection, which can facilitate their use in routine clinical practice. It is also noteworthy that targeting YY1 binding partners, such as p300 and BRD4, can also lead to therapeutic effects that are similar to targeting the YY1 protein itself [79,88,89,275,276,277]. Further investigation into these alternative approaches could offer new possibilities for developing more effective YY1-targeted therapies.

**Figure 3:**
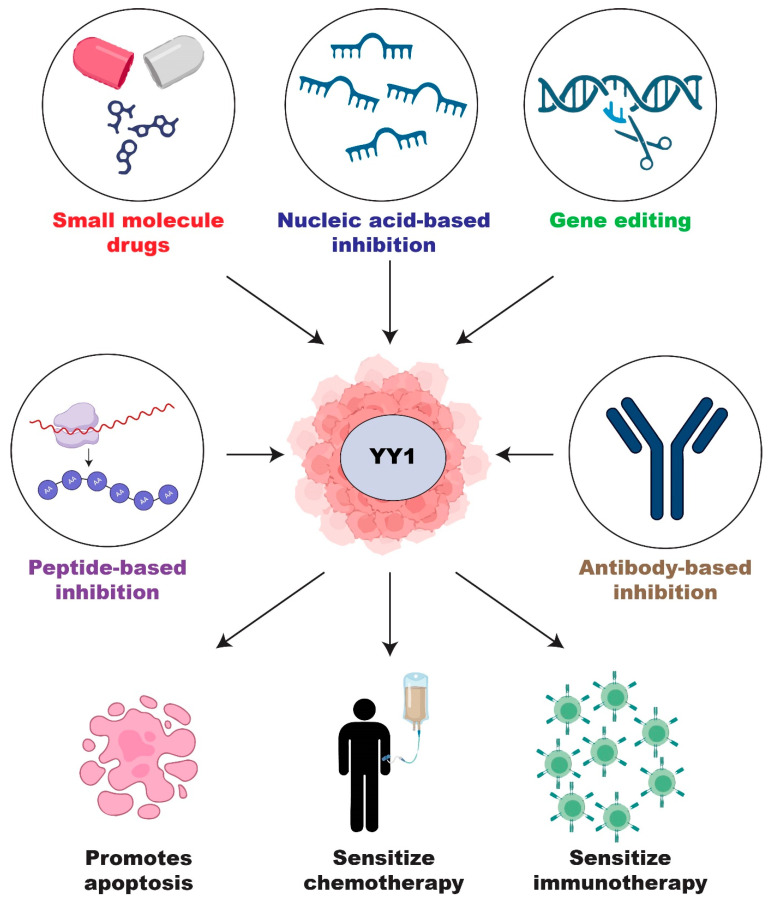
Overview of the current strategies and potential of targeting YY1 in antitumor therapy.

Overall, YY1 has garnered extensive research interest for its role in gene regulation in tumor cells, exhibiting both activation and repression capabilities. Its involvement in various cancer hallmarks highlights its potential as a target for antitumor therapy. However, challenges remain in terms of drug specificity, efficacy, and delivery. To augment the success of YY1-targeted therapies, strategies such as combination therapies, alternative drug delivery methods, and the identification of treatment response biomarkers may be employed to improve the efficacy and clinical translation of YY1-targeted therapies. Nevertheless, targeting YY1 is a promising antitumor strategy, and the innovative approach mentioned in this review could lead to successful treatments and improved patient outcomes in the future (Figure 3).

## Figures and Tables

**Table 1:** Expression of YY1 in various cancers.

Cancer Types	YY1 Expression Level	Prognosis	Refs.
Bladder	Upregulated	Poor	[33,34]
Breast	Upregulated	Poor	[35]
Cervical	Upregulated	Poor	[36,37]
Colon	Upregulated	Poor	[38,39,40,41]
Esophageal	Upregulated	Poor	[42]
Gastric	Upregulated	Poor	[43,44]
Glioma	Upregulated	Poor	[45,46,47]
Hodgkin lymphoma	Upregulated	n/a	[48]
Leukemia	Upregulated	Poor	[49,50,51]
Liver	Upregulated	Poor	[52]
Lung	Upregulated	Poor	[27,53]
Melanoma	Upregulated	Poor	[51,54,55,56]
Multiple myeloma	Upregulated	Poor	[57]
Nasopharynx	Downregulated	Good	[58]
Non-Hodgkin lymphoma	Upregulated	Poor	[59,60,61,62]
	Downregulated	Good	[63]
Osteosarcoma	Upregulated	Poor	[32]
Ovarian	Upregulated	Poor	[23,64,65]
Pancreatic	Upregulated	Poor	[66]
	Upregulated	Poor	[67]
Renal	Upregulated	Poor	[68]
Sarcoma	Upregulated	n/a	[69]
	Upregulated	Poor	[32]
Testicular seminoma	Upregulated	Poor	[70,71]
Thyroid	Upregulated	Poor	[72,73,74]

n/a: not available.

**Table 2:** Transcription factor YY1 regulates the hallmarks of cancer.

Target	Pathway	Hallmarks	Refs.
AKT	YY1/mTORC2/AKT	Evading apoptosis; limitless replicative potential; sustained angiogenesis; tissue invasion and metastasis	[74,94]
APC	miR-193a-5p/YY1/APC	Limitless replicative potential	[95]
Atg5	YY1/TFEB/Atg5-Atg12-Atg16	Evading apoptosis (by evading autophagy)	[56]
Beclin1	YY1/TFEB/Beclin1	Evading apoptosis (by evading autophagy)	[56]
Bim	YY1/RelA/Bim	Evading apoptosis; limitless replicative potential	[96]
CDKN2A	YY1/HDACs/CDKN2A	Evading apoptosis	[97,98]
CDKN3	YY1/CDKN3/MdM2/p53/p21	Limitless replicative potential; tissue invasion and metastasis	[99]
c-Myc	YY1/c-Myc	Deregulated metabolism; evading apoptosis; genome instability; limitless replicative potential; tissue invasion and metastasis	[87,100]
COX2	YY1/COX2/PG	Evading immune system	[101,102]
CXCR4	CXCR4/YY1/VEGF	Sustained angiogenesis; tissue invasion and metastasis	[103]
CXCR4	SDF-1α/CXCR4/YY1/let-7a	Evading apoptosis; evading immune system	[104]
DEK	YY1/DEK/HIF-1α/VEGF	Sustained angiogenesis	[105]
DEK	YY1/NF-Y/DEK	Limitless replicative potential	[106]
DR5	YY1/DR5/TRAIL/NF-κB	Evading apoptosis; evading immune system	[107,108]
DTDST	NF-κB/YY1/PRC2-EZH2/ DTDST	Evading immune system; limitless replicative potential; tissue invasion and metastasis	[109]
CDH1	YY1-PRMT7-HDAC3/H3K4me3/CDH1	Tissue invasion and metastasis	[110]
EGFR	mir-34a/YY1/EGFR	Limitless replicative potential	[111]
EGFR	MCT1/YY1/EGFR/MnSOD	Deregulate metabolism; evading apoptosis	[112]
ERBB2	YY1/AP-2α/ERBB2	Sustained angiogenesis; tissue invasion and metastasis	[113]
Fas	miR27a/ZBTB10/Sp/YY1/ ERBB2	Limitless proliferative potential	[75]
G6PD	YY1/G6PD/PPP/r5p	Deregulated metabolism	[85]
GLUT3	YY1/GLUT3	Deregulated metabolism; limitless replicative potential	[91]
HIF-1α	YY1/HIF-1α/GLUT1-GLUT3	Deregulated metabolism; evading apoptosis	[114]
HIF-1α	YY1/HIF-1α/VEGF & TGF-α	Sustained angiogenesis; tissue invasion and metastasis	[84,103]
HIF-1α	YY1/HIF-1α/CA9	Evading immune system; tissue invasion and metastasis	[84,115,116]
HIF-1α	YY1/HIF-1α/PGK	Evading apoptosis; deregulated metabolism; sustaining proliferative signaling	[84,117]
hnRNPM	YY1/hnRNPM/CD44	Tissue invasion and metastasis	[118]
HPV18	YY1-CTCF/HPV18	Insensitivity to anti-growth signals; limitless replicative potential	[119]
IL6	YY1/IL6/STAT3/PD-L1	Evading immune system	[22]
KLF4	YY1/KLF4/p53	Evading apoptosis	[120]
KLF5	YY1/KLF4/p21	Limitless replicative potential	[120]
KLF6	YY1/KLF4/c-Myc	Deregulated metabolism; evading apoptosis; genome instability; limitless replicative potential; tissue invasion and metastasis	[87,120]
KLF7	YY1/KLF4/cyclin D2	Limitless replicative potential	[120]
MAP1LC3B	YY1/TFEB/MAP1LC3B	Evading apoptosis	[56]
miR-125a	RYBP/YY1/pri-miR-125a	Evading apoptosis, evading immune system	[121]
miR-195	miR-195/Smurf2YY1/VEGFA/ Snail1	Tissue invasion and metastasis	[122]
miR-30a	YY1/miR-30a/ATG5 & Beclin1	Evading apoptosis	[123]
miR-372	YY1/mIR-372/SQSTM1	Evading apoptosis	[124]
miR-9	YY1/EZH2/H3K27me3/miR9/ NF-κB1	Evading apoptosis; tissue invasion and metastasis	[30]
p21	YY1/BCCIP/p53re/p21	Evading apoptosis; limitless replicative potential	[90]
p53	YY1/BCCIP/p53re/p21	Evading apoptosis; limitless replicative potential	[90]
p53	YY1/MDM2/p53	Evading apoptosis	[89]
p53	p14ARF/YY1/Hdm2/p53	Evading apoptosis; insensitivity to anti-growth signals	[88]
p53	YY1/TIGAR/PDK2/PFK-1	Deregulated metabolism; evading apoptosis	[125]
p53	YY1/p300/MDM2/p53	Evading apoptosis	[88]
p53	Smurf2/YY1/p53	Evading apoptosis; evading immune system	[126,127]
p73	YY1/E2F1/p73	Evading apoptosis; insensitivity to anti-growth signals	[128]
PGC-1β	YY1/PGC-1β/MCAD & LCAD	Deregulated metabolism	[92]
RelB	YY1/RelB/p65 & p50	Evading apoptosis; evading immune system	[46]
RYBP	YY1/miR-9/RYBP/SP1	Evading apoptosis; insensitivity to anti-growth signals; tissue invasion and metastasis	[129]
RYBP	RYBP/YY1/E2F6/Mae1 or Staq3 or Smc1β	Insensitivity to anti-growth signals; limitless replicative potential	[130]
RYBP	RYBP/YY1/E2F2 or E2F3/CDC7	Genome instability; insensitivity to anti-growth signals; limitless replicative potential	[130]
ST6GalNAc6	YY1/PRC2/EZH2/H3K27me3/ DTDST/ST6GalNAc6	Evading immune system; genome instability	[97,109]
TPPP	YY1/TPPP/p38/MAPK	Evading apoptosis; sustained angiogenesis; tissue invasion and metastasis	[131]
TPPP	YY1/TPPP/PI3K/AKT	Sustained angiogenesis; tissue invasion and metastasis	[131]
VEGF	CXCR4/YY1/VEGF	Sustained angiogenesis; tissue invasion and metastasis	[103]
VEGF	YY1/VEGFA/VEGFR2	Evading apoptosis; sustained angiogenesis	[132]
VEGFB	CXCR4/YY1/VEGFB	Sustained angiogenesis; tissue invasion and metastasis	[103]

## Data Availability

Not applicable.
